# GPER in murine astrocytes and mural cells promotes neurovascular coupling and Aβ clearance

**DOI:** 10.21203/rs.3.rs-10359258/v1

**Published:** 2026-07-30

**Authors:** Mengjiao Xu, Ke Chen, Meimei Wu, Huashan Gong, Ping Luo, Jing Wang, Weifang Rong

**Affiliations:** 1 Department of Gastroenterology, Songjiang Research Institute, Songjiang Hospital Affiliated to Shanghai Jiao Tong University School of Medicine, Shanghai 201600, China; 2 Department of Anatomy and Physiology, Shanghai Jiao Tong University School of Medicine, Shanghai 200025, China; 3 School of Basic Medical Science and Key Laboratory of Craniocerebral Diseases of Ningxia Hui Autonomous Region, Ningxia Medical University, Yinchuan 750004, China.

**Keywords:** GPER, neurovascular unit, Alzheimer’s disease, Aβ clearance, sexual dimorphism, astrocytes, mural cells, glymphatic system

## Abstract

Alzheimer’s disease (AD) affects aged women more than men. The precipitous drop in sex hormone levels in postmenopausal women likely contributes to such sexual dimorphism, with emerging evidence implicating the G protein-coupled estrogen receptor (GPER) as a modulator of AD pathology and cognitive impairments. However, the underlying mechanism remains unknown. GPER is distinctively expressed in key components of the neurovascular unit (NVU), namely astrocytes and mural cells (specifically, pericytes). Using GPER-knockout (GPER-KO) and APP/PS1 transgenic AD mouse models, here we show that GPER deficiency exacerbates Aβ deposition, impairs spatial and working memory, and disrupts neurovascular coupling. GPER activation in pericytes reinforces *Lrp1*-dependent endocytic uptake of Aβ. In astrocytes, GPER appears to be essential in regulation of AQP4 polarity, gap junction protein expression and reactivity. Furthermore, GPER interacts directly with Aβ to form a protein complex and loss of GPER leads to astrocytic dysfunction, reduced pericyte coverage, and impaired glymphatic clearance. Our findings reveal GPER as a critical regulator of NVU function and Aβ proteostasis, providing a mechanistic basis for AD sexual dimorphism, further supporting GPER as a potential target for sex-specific therapeutic strategies.

## Graphical Abstract

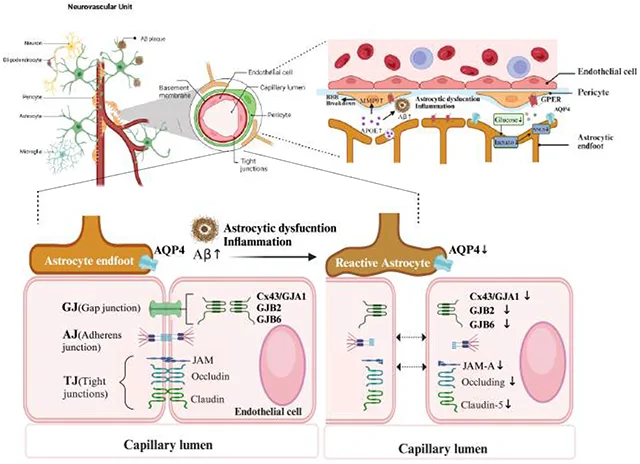

## Background

Alzheimer’s disease (AD) is characterized by the pathological accumulation of amyloid-beta (Aβ) peptides and neurofibrillary tangles, which drive progressive neuronal dysfunction^[1, 2]^. While historically viewed through a “neuron-centric” lens, the pathogenesis of AD is increasingly recognized as a systemic failure of the neurovascular unit (NVU), giving rise to the “neurovascular hypothesis” of neurodegeneration^[3]^. Epidemiological evidence highlights a significant sexual dimorphism in AD, with women comprising approximately two-thirds of all cases and exhibiting more rapid cognitive decline post-menopause^[4, 5]^. This phenomenon is closely associated with the precipitous decline in circulating 17β-estradiol (E2) levels, which can plummet by up to 90% during the perimenopausal transition^[6–8]^. Such findings suggest that the loss of estrogen signaling is a critical driver of the heightened susceptibility to AD in women^[9]^.

Estrogen exerts its biological effects via classical nuclear receptors (ERα and ERβ) and the membrane-associated G protein-coupled estrogen receptor (GPER/GPR30)^[10]^. Unlike nuclear ERs that mediate long-term genomic effects, GPER triggers rapid non-genomic signaling cascades and is highly enriched in cognitive hubs such as the prefrontal cortex and hippocampus^[11]^. Recent epigenome-wide association studies have identified GPER DNA methylation as a significant predictor of accelerated cognitive decline and tau pathology in women, positioning GPER as a central player in AD etiology^[12, 13]^. However, despite its established importance, the cell-type-specific mechanisms by which GPER preserves brain health—particularly within the non-neuronal compartments of the NVU—remain largely elusive.

Current paradigms suggest that Aβ accumulation stems from an imbalance between its production and clearance^[14, 15]^. The efficient removal of Aβ from the brain parenchyma is a highly coordinated process involving the blood-brain barrier (BBB) and the glymphatic system, both of which are governed by the NVU^[16, 17]^. Emerging evidence underscores that astrocytic endfeet and pericytes are the primary controllers of glymphatic flux and blood flow, respectively^[16, 18]^. Our previous findings revealed that GPER is uniquely positioned within the NVU^[19]^, showing robust expression not only in neurons but also in pericytes and astrocytes. This spatial distribution suggests that GPER may function as a master regulator of neurovascular homeostasis. Nevertheless, whether GPER coordinates pericyte-mediated phagocytosis and astrocytic glymphatic flux to maintain neurovascular coupling (NVC) and Aβ homeostasis remains an open question. In this study, we aimed to investigate the potential role of GPER in astrocytic and pericytic functions within the AD neurovascular niche and attempted to determine its underlying regulatory mechanisms governing neurovascular coupling and amyloid clearance.

## Materials and methods

### Animals and genotyping

GPER-knockout (GPER-KO) mice (C57BL/6J background) were generated via the CRISPR/Cas9 system by BIORAY (Shanghai, China), harboring a 17-bp deletion in the Gper gene (Gene ID: 76854)^[20]^. Genotyping was confirmed by PCR analysis using the following primers flanking the deletion site: forward 5′-AACAGGCTCCCAGGACGAT-3’;and reverse, 5′-ATCCAGGTGAGGAAGAAGACG-3’. PCR products were resolved by agarose gel electrophoresis to distinguish between WT and GPER-KO alleles. Wild-type (WT) littermates served as controls.

APP/PS1 mice were kindly provided by Prof. Nanjie Xu (Shanghai Jiao Tong University, Shanghai, China). To generate the conditional knockout model (APP/PS1;GFAP^cre^-GPER^fl/fl^), APP/PS1 mice were crossed with GFAP^cre^-GPER^fl/fl^ mice. GPER^fl/fl^ and APP/PS1;GPER^fl/fl^ littermates were utilized as corresponding controls.

All mice were maintained on a C57BL/6J background. Experiments were performed on both male and female mice aged 6-12 months (20–30g). Age- and sex-matched mice were randomly assigned to experimental groups for behavioral and pathological analyses. Animals were housed in a specific pathogen-free (SPF) facility under standard conditions (22 ± 2 °C; 50% ± 10% humidity; 12-h light/dark cycle) with ad libitum access to food and water. All animal procedures were approved by the Institutional Animal Care and Use Committee (IACUC) of Shanghai Jiao Tong University School of Medicine and were conducted in strict accordance with the National Institutes of Health (NIH) Guide for the Care and Use of Laboratory Animals.

### Barnes maze test

Spatial reference memory was evaluated using the Barnes maze test. The apparatus comprised an elevated circular platform (100 cm in diameter, 100 cm above the floor) featuring 18 equally spaced holes along the periphery, with one hole connected to a target escape box. Fixed extra-maze visual cues were provided in the testing room to facilitate spatial orientation. Mice were acclimatized to the testing room for 30 min prior to each session. The training regimen consisted of two trials per day for five consecutive days. At the onset of each trial, mice were placed in the center of the platform within an opaque starting cylinder for 5 s to ensure random orientation. Upon removal of the cylinder, mice were allowed to explore the maze for a maximum of 180 s to locate the target box. Mice that failed to enter the box within 180 s were gently guided to the target and allowed to remain there for 30 s. To eliminate intra-maze olfactory cues, the platform surface was thoroughly cleaned with 70% ethanol between trials. The maze was randomly rotated between sessions, while maintaining the fixed spatial relationship between the target box and extra-maze cues. Spatial learning and memory were quantified by recording escape latency, time spent in the target quadrant, and error frequency (defined as nose-pokes into non-target holes) on the final training day. All behavioral data were recorded and analyzed using an automated video tracking system (EthoVision XT, Noldus Information Technology, Wageningen, The Netherlands).

### Y-maze test

Working memory was assessed using the spontaneous alternation Y-maze task. The maze consisted of three identical arms (labeled A, B, and C) at 120° angles. Mice were placed in the center of the maze and allowed to explore the arms freely for 5 min. An alternation was defined as consecutive entries into three different arms (e.g., ABC, BCA, or CAB). The total number of arm entries and the number of successful alternations were recorded. The percentage of spontaneous alternation was calculated as: [(number of alternations) / (total arm entries - 2)] × 100. To ensure the reliability of the working memory assessment, mice with fewer than 8 total arm entries were excluded from the analysis. The maze was thoroughly cleaned with 70% ethanol between trials to eliminate olfactory cues. All behavioral parameters were recorded and analyzed using an automated video tracking system (EthoVision XT, Noldus Information Technology, Wageningen, The Netherlands).

### Laser Speckle Contrast Imaging (LSCI) and Neurovascular Coupling Assessment

Cerebral blood flow (CBF) dynamics were assessed in 12-month-old WT, GPER-KO, and APP/PS1 mice using a laser speckle contrast imaging (LSCI) system (RFLSI ZW; RWD Life Science, Shenzhen, China). Mice were anesthetized via an intraperitoneal injection of 1% sodium pentobarbital (70 mg/kg). During the entire procedure, body temperature was maintained at 37 ± 0.5 °C using a feedback-controlled heating pad. After shaving the scalp and disinfecting the skin with 70% ethanol, a midline incision was made to expose the skull. CBF was measured through the intact skull.

The imaging system was configured with a frame rate of 50 fps and a pseudo-color image resolution of 1032 × 772 pixels. A sliding algorithm was employed to enhance the temporal resolution and signal-to-noise ratio of the LSCI data, allowing for the precise detection of rapid hemodynamic changes during stimulation. Data were acquired over a 2.5-min experimental period. The region of interest (ROI) was consistently positioned over the primary somatosensory cortex (S1) contralateral to hindpaw stimulation, identified by stereotaxic coordinates relative to bregma (AP: −0.5 to −1.0 mm, ML: +1.5 to 2.2 mm).

During the acquisition, the S1 cortex was monitored during a 30-s hindpaw mechanical stimulation paradigm to induce functional hyperemia. To ensure consistency of the mechanical stimulus, the pressure applied by the miniature clip was monitored using a micro-pressure gauge (Chengdu Jingjuda Electronic Technology Co., Ltd., Chengdu, China), confirming that the stimulation intensity remained constant (mean force: 33 N) . CBF values within the selected ROI were recorded for each mouse. The relative changes in CBF were calculated as a percentage of the baseline resting value and plotted as hemodynamic waveforms to evaluate neurovascular coupling integrity and vascular responsiveness.

### In vivo Two-Photon Imaging and Hemodynamic Analysis

To evaluate neurovascular coupling (NVC) further, in vivo two-photon laser scanning microscopy (TPLSM) imaging (FV31S-SW; Olympus, Tokyo, Japan) was performed on 12-month-old WT and GPER-KO mice. Mice were initially anesthetized with an intraperitoneal injection of 1% pentobarbital sodium (70 mg/kg) and secured in a stereotaxic frame (Kopf Instruments, Tujunga, CA, USA). Anesthesia was subsequently maintained with isoflurane (1.0%-1.5% in a gas mixture of O2 and air) via a nose cone. A cranial window was prepared by thinning the skull over the hindlimb primary somatosensory cortex (S1HL; centered at AP: −1.0 mm, ML: +1.5 mm relative to bregma) using a micro-drill (Meinaite, Germany). Given the focus on capillary imaging, the skull was thinned sufficiently to allow high-quality visualization without the use of a coverslip. Body temperature was maintained at 37 ± 0.5 °C using a feedback-controlled heating pad.

The vasculature was labeled via a retro-orbital injection of 20 kDa FITC-dextran (0.1 mL, 10 mg/mL; Sigma-Aldrich, USA). Imaging was conducted using a two-photon microscope with an excitation wavelength of 910 nm. After identifying regions responsive to hindpaw stimulation via high-resolution imaging (512 × 512 pixels, 10.0 μs/pixel), electrical stimulation was applied to the contralateral hindpaw using custom-made electrodes (Shanghai Jiayi Factory, Shanghai, China). The stimulation parameters were set to 10 V, 10 Hz frequency, and 5 ms pulse duration for a total duration of 10 s. Line-scan imaging was performed along the longitudinal axis of individual capillaries at a high sampling rate (Sampling Speed: 10.0 us/pixel). Space-time images were generated, where red blood cells (RBCs) appeared as dark shadows against the fluorescently labeled blood plasma. The slope of the resulting streaks is inversely proportional to the RBC velocity. Velocity data were processed using a custom MATLAB algorithm^[21]^ (MathWorks, Natick, MA, USA), followed by low-pass (1 Hz cutoff) and notch (0.5 Hz) filtering to eliminate cardiac and respiratory artifacts. Automated smoothing was achieved using a 1-s boxcar filter via a custom-written protocol in Igor Pro (WaveMetrics, Lake Oswego, OR, USA)^[21]^. Baseline velocity was defined as the average velocity during the 10 s preceding the stimulation. The LS-PIV MATLAB code is available in the Supporting Information (Text S1).

### Primary Cell Cultures

Primary cortical astrocytes were isolated from postnatal day 0 (P0, C57BL/6J) mice following a modified serum-based protocol derived from the classic McCarthy and de Vellis method^[22]^. Briefly, cerebral cortices were dissected on ice, mechanically dissociated, and passed through cell strainers to yield a single-cell suspension.The cells were purified via differential centrifugation and seeded at an initial density of approximately 1×10^5^ cells/cm^2^ in 75-cm^2^ flasks containing DMEM (Servicebio, Wuhan, China) supplemented with 10% fetal bovine serum. Upon reaching ~60% confluency, astrocytes were harvested and replated onto poly-D-lysine (PDL)-coated coverslips. Cultures were subsequently fixed with 4% paraformaldehyde (PFA) at room temperature for immunocytochemical staining and imaging. Primary brain vascular pericytes were purchased from Shanghai Haixing Biosciences Co., Ltd. (Shanghai, China).

### Immunocytochemistry

Following fixation with 4% paraformaldehyde (PFA) at room temperature for 15 min, cells were washed three times with PBS and permeabilized/blocked in PBS containing 10% normal donkey serum (NDS) and 1% Triton X-100 for 1 h at room temperature to minimize non-specific binding. Samples were then incubated overnight at 4 °C with primary antibodies against PDGFRβ (goat; Bio-Techne, Minneapolis, MN, USA), Lrp1 (rabbit; Abcam, Cambridge, UK), and LAMP1 (rabbit; Abcam). After three washes with PBS, cells were incubated with corresponding species-specific fluorophore-conjugated secondary antibodies at room temperature for 2 h in the dark. Detailed information regarding all primary and secondary antibodies (including catalog numbers and dilution ratios) is provided in Supplementary Table S1. Nuclei were counterstained with DAPI (Boster, Wuhan, China) for 10 min. Fluorescence images were acquired using an Olympus FV3000 confocal microscope (Olympus, Tokyo, Japan). For quantitative analysis, the mean fluorescence intensity (MFI) and the number of positive cells were quantified using ImageJ software (NIH, Bethesda, MD, USA). Data were collected from at least 3 independent biological replicates (derived from n=3 mice), with a minimum of 4 random fields of view captured per sample.

### FITC-Aβ Uptake Assay

Primary murine brain vascular pericytes were treated with the GPER agonist G1 μmol/L or 17β-estradiol (E2, 1 μmol/L) for 24 h. The cells were then incubated with FITC-conjugated Aβ_1–42_ (FITC-Aβ, 1 μg/mL) for 4 h at 37°C. Following incubation, cells were fixed with 4% paraformaldehyde (PFA) at room temperature for 15 min, washed with PBS, and subjected to immunocytochemical staining with a rabbit anti-LAMP1 primary antibody to label lysosomes, as described in the “Immunocytochemistry” section. Detailed antibody information is provided in Supplementary Table S1. Fluorescence images were captured using an Olympus FV3000 confocal microscope (Olympus, Tokyo, Japan). The intracellular mean fluorescence intensity (MFI) of FITC-Aβ and LAMP1 immunoreactivity were quantified using ImageJ software (NIH, Bethesda, MD, USA) to evaluate phagocytic efficiency and lysosomal degradation capacity, respectively.

### Tissue preparation and Immunohistochemistry

Mice were anesthetized with 2 % pentobarbital sodium and transcardially perfused with cold PBS followed with cold 4 % paraformaldehyde (Epizyme, Shanghai, China). Then, intact brains were collected and stored in 4 % paraformaldehyde at 4°C overnight. Brain samples were subsequently dehydrated by 30 % sucrose solution at 4°C for 48 h. Brains were serially sectioned on cryostat (Leica) at a thickness of 35 μm optimized for individual staining. All IF staining was performed on free floating sections. Sections were blocked in phosphate-buffered saline solution containing 1% Triton X-100, 10% normal donkey serum at room temperatureat for 1 h. Sections were then incubated overnight at 4°C with the following primary antibodies diluted in blocking buffer: Anti-β-amyiod Mouse 1-16 (6E10, Biolegend), Alexa Flour^£^ 647 Anti-β-amyiod Mouse(Biolegend), AQP4-Rat (Oasis), GFAP-guinea pig (Oasis), p-Tau-rabbit (Immunoway), GPER-rabbit (abclonal), Alexa Flour^®^ 594 CD31 (Biolegend), CD31-goat (R&D), Gja1-rabbit (abclonal), Lrp1-rabbit (Abcam) and goat anti-PDGFRβ (Bio-Techne) were utilized. Following primary antibody incubation, sections were washed three times with PBS and incubated with species-specific Alexa Fluor-conjugated secondary antibodies (1:500; abcam) for 2 h at room temperature in the dark. Nuclei were counterstained with DAPI (0.5 μg/mL) included in the secondary antibody solution. Finally, sections were mounted onto gelatin-coated slides and coverslipped with anti-fade mounting medium.

### Slide Scanning and Confocal Imaging

Full brain images were acquired using automated, slide-scanning microscopy (VS200, Olympus) with a ×20 objective. For AQP4 quantification along various vessel diameters, images were acquired on confocal microscopy (Leica TCS Stellaris8 STED) with a ×20 and ×63 objective. To maximize the uniformity of quantifications across images, all laser intensities were selected to minimize signal outside of the dynamic range of the detector across all samples imaged. For 3D volumetric analysis and spatial quantification, high-resolution Z-stack datasets were imported into Imaris software (Bitplane, Oxford Instruments). The “Surface” and “Spots” creation wizards were utilized to generate 3D renderings of Aβ aggregates, astrocytic endfeet, and vascular networks based on absolute intensity thresholds. To assess the spatial relationship between GPER and amyloid pathology, the “Colocalization” module was employed to calculate the voxel-wise overlap coefficients and the volume of Aβ sequestered within specific cellular domains (e.g., GFAP^+^ astrocytes or PDGFRβ^+^ pericytes).

### Transmission Electronic Microscopy

Mice were deeply anesthetized and perfused. The brains were rapidly harvested on ice. Tissue blocks (approximately 1 mm^3^) containing the somatosensory cortex and hippocampus were dissected and immediately immersed in electron microscopy fixative at 4°C overnight. Samples were submitted to Pinuofei Biotechnology for transmission electron microscopy (TEM) imaging to characterize tissue composition and ultrastructure. Following image acquisition, data were analyzed using digital imaging software.

### Real-Time Quantitative RT-PCR

qRT-PCR was performed as previously described. Total RNA was extracted using TRIzol reagent. First-strand cDNA was synthesized from 1 μg of the extracted RNA using a cDNA synthesis kit from TIANGEN. qRT-PCR was performed on an Agilent Mx3005P RT-PCR system. All the primers used are listed in Table S1 (Supporting Information).

### Western Blotting

Protein samples were boiled for 10 min at a temperature between 95 and 100 °C, separated by SDS-PAGE, transferred to a membrane, and incubated with the primary antibody at 4 °C overnight. The next day, after washing three times, the membrane was incubated with horseradish peroxidase (HRP)-conjugated secondary antibody at room temperature for 1 h. Finally, an enhanced chemiluminescent (ECL) substrate was added to the membrane, and the protein signal was detected using autoradiography film (RWD).

### Image analysis and quantification

All image analyses were performed using Fiji (ImageJ; NIH, Bethesda, MD, USA) and Imaris software. For representative figures, uniform and linear adjustments were applied to pixel intensities; however, all quantitative analyses were executed on raw, unaltered images. Behavioral data from Barnes maze and Y-maze tests were recorded and analyzed using EthoVision XT (Noldus). For *in vivo* two-photon imaging, raw velocity traces were smoothed using a 1-s boxcar filter in Igor Pro (WaveMetrics) to eliminate cardiac and respiratory artifacts, and RBC velocity was calculated via custom MATLAB (MathWorks) scripts based on line-scanning particle image velocimetry (LS-PIV).

For immunofluorescence quantification, regions of interest (ROIs) were defined in a blinded manner, followed by automated quantification using custom Fiji scripts. Perivascular AQP4 polarization was quantified by measuring the fluorescence intensity ratio between the perivascular space and the parenchymal compartment. Amyloid burden (6E10 staining) expression was quantified as the percentage of positive area within the cortex and hippocampus. Capillaries were defined as microvessels with a diameter of < 10 μm. A small fraction of poorly focused images (1%–2%) were excluded from the analysis, and integrated density was calculated across the remaining sections.

### Statistical analysis

Statistical analyses were performed using GraphPad Prism 10.1.2 (GraphPad Software, San Diego, CA, USA). Data are presented as the mean ± SEM. Comparisons between two groups were performed using two-tailed unpaired Student’s t-tests. For multiple group comparisons across different genotypes and ages, two-way ANOVA was primarily employed to evaluate the main effects and interactions, followed by Tukey’s or Sidak’s post hoc tests. In instances where data were missing or to account for within-subject correlations, a mixed-effects model was applied. All P-values were adjusted for multiple comparisons, and differences were considered statistically significant at P < 0.05. Detailed statistical parameters, including sample sizes (n), specific tests used, and exact P-values, are provided in the corresponding text and figure legends.

## Results

### GPER deficiency exacerbates age- and sex-dependent cognitive decline in mice

**Figure 1.**
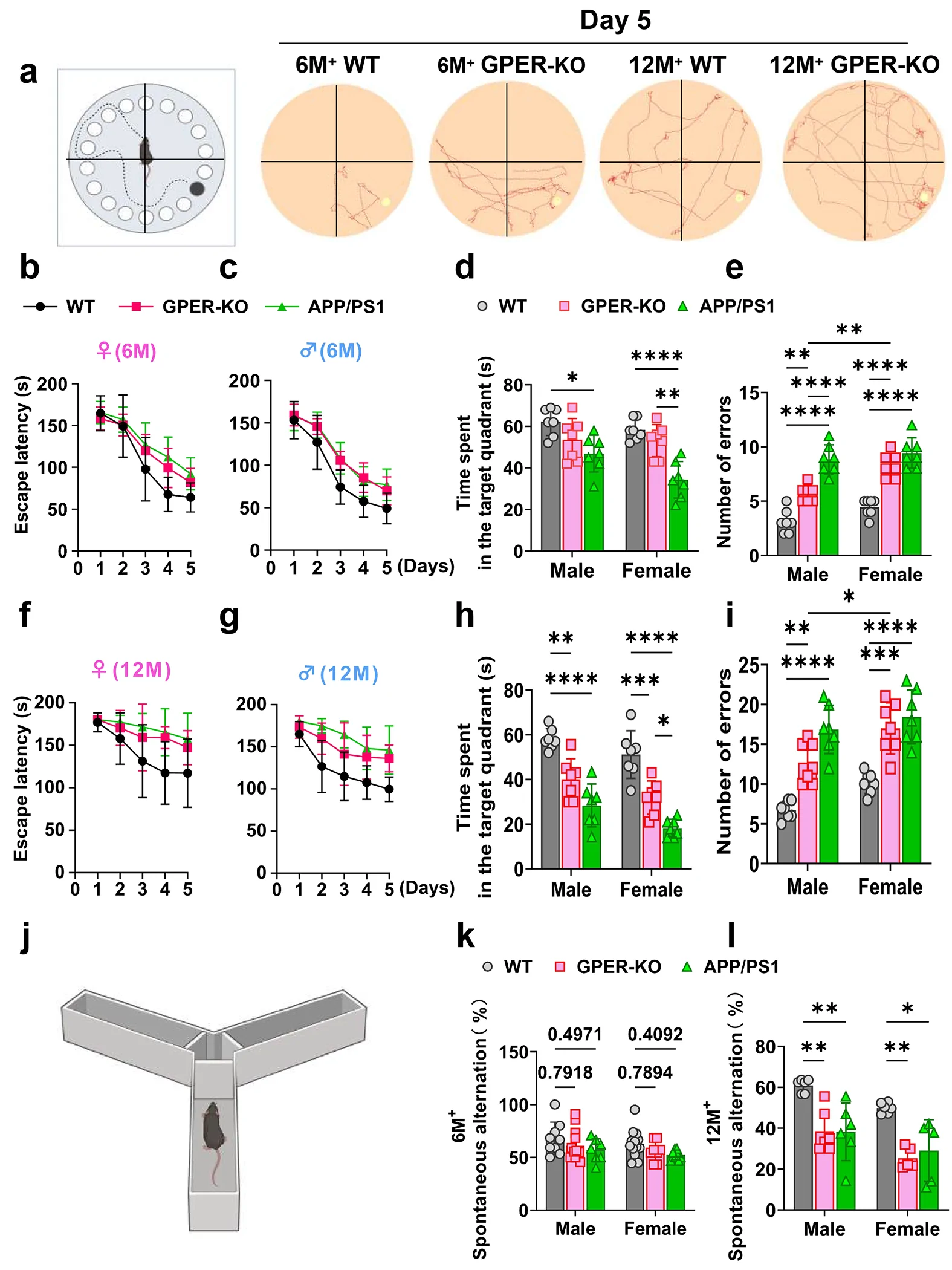
GPER deficiency exacerbates age- and sex-dependent spatial and working memory deficits in mice. (a) Schematic diagram of the Barnes maze apparatus (left) and representative tracking paths of WT, GPER-KO, and APP/PS1 mice during the probe trial (right). (b, c) Escape latency of 6-month-old (6M^+^) female (b) and male (c) mice of the indicated genotypes during the 5-day training phase. (d, e) Probe trial performance of 6M^+^ mice, indicating the time spent in the target quadrant (d) and the number of errors made before locating the escape tunnel (e). (f, g) Escape latency of 12-month-old (12M^+^) female (f) and male (g) mice of the indicated genotypes during the 5-day training phase. (h, i) Probe trial performance of 12M^+^ mice, showing the time spent in the target quadrant (h) and the number of errors (i). (j) Schematic illustration of the Y-maze spontaneous alternation test. (k, l) Spontaneous alternation rates (%) in the Y-maze for 6M^+^(k) and 12M^+^ (l) female and male mice across different genotypes. Data are presented as mean ± SEM (n=22 for WT; n=20 for GPER-KO; n=20 for APP/PS1). Statistical significance was determined by two-way ANOVA followed by Tukey’s *post hoc* test for multiple comparisons. *P<0.05, **P<0.01, ***P<0.001 compared with WT.

To evaluate the impact of GPER on cognitive maintenance, we utilized age-matched cohorts of wild-type (WT, n=**22**), GPER-knockout (GPER-KO, n=**20**), and APP/PS1 transgenic (n=**20**) mice on a C57BL/6J background, with balanced numbers of males and females. We performed Barnes maze (Figure 1a–i) and Y-maze tests (Fig. 1j–l) on 6- and 12-month-old (6M^+^ and 12M^+^) mice across these three genotypes to assess hippocampal-dependent spatial and working memory^[23, 24]^.

The Barnes maze test revealed a progressive, age-dependent spatial memory deficit in GPER-KO mice. Representative tracking plots demonstrated that while WT mice utilized efficient search strategies, GPER-KO mice exhibited highly disorganized trajectories (Fig. 1a). During the training phase, both female and male GPER-KO mice exhibited significantly prolonged escape latencies compared to their sex-matched WT counterparts by the final training day, at both 6M^+^ (Fig. 1b, c) and 12M^+^ of age (Fig. 1f, g). Notably, the spatial learning deficit in GPER-KO mice represented an intermediate phenotype, being less severe than that in APP/PS1 mice but significantly worse than in WT controls. Female mice across all genotypes consistently demonstrated poorer performance than males, indicating a baseline sexual dimorphism in this task^[25, 26]^.

In the probe trial, 6M^+^ GPER-KO mice exhibited a moderate reduction in time spent in the target quadrant and an increased number of errors compared to WT mice (Fig. 1d–e). By 12M^+^, this memory retention deficit worsened drastically. Both female and male 12M^+^ GPER-KO mice spent significantly less time in the target quadrant (Females: 31.86±4.52 s vs. WT: 51.14±5.12 s, P=0.0008; Males: 40.09±4.11 s vs. WT: 58.14±4.89 s, P=0.0019; Figure 1h) and committed a high frequency of errors (Fig. 1i), performing at levels statistically comparable to the severely impaired APP/PS1 mice. Notably, female GPER-KO mice consistently demonstrated poorer performance than their male counterparts, indicating a heightened vulnerability.

The Y-maze test further confirmed working memory impairment in GPER-KO mice (Fig. 1j). At 6M^+^, GPER-KO mice showed a mild reduction in spontaneous alternation rates compared to WT controls (Fig. 1k). However, at 12M^+^, GPER-KO female mice exhibited a catastrophic decline in spontaneous alternation, dropping to levels equivalent to or even slightly lower than those of APP/PS1 females. A significant, albeit slightly less severe, deficit was also observed in 12M^+^ GPER-KO males (Fig. 1l).

Taken together, these data establish GPER as a critical determinant of cognitive health during aging. GPER-KO mice exhibit an intermediate cognitive decline phenotype between WT and APP/PS1 mice, with females being more severely affected, mirroring the sexual dimorphism commonly observed in clinical Alzheimer’s disease.

### GPER deficiency promotes Aβ accumulation

**Figure 2.**
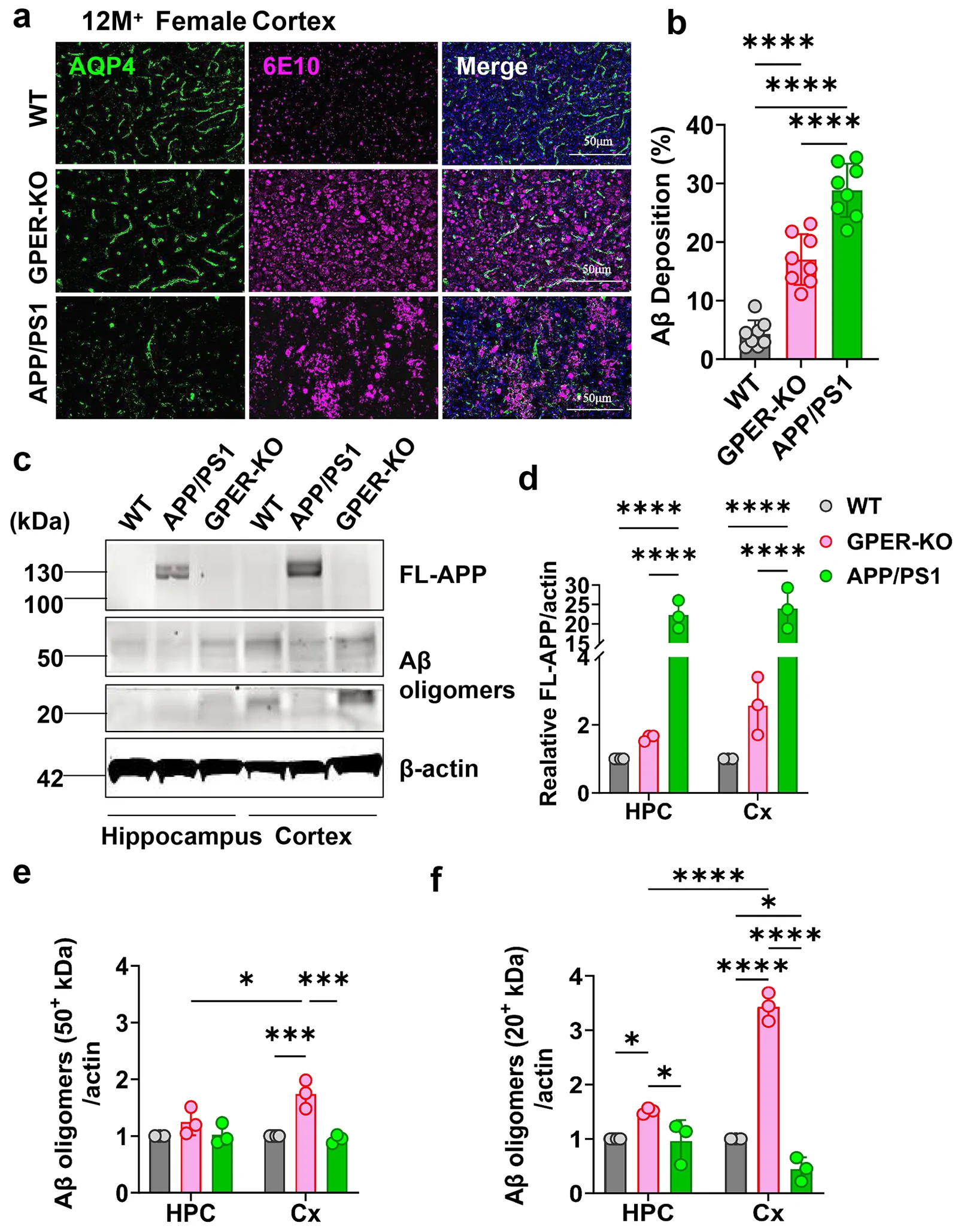
GPER deficiency promotes amyloid deposition in 12-month-old female mice. (a) Representative confocal immunofluorescence images showing AQP4 (green), Aβ (6E10, magenta), and nuclei (DAPI, blue) in the cortex of WT, GPER-KO, and APP/PS1 mice. Scale bar = 50 μm. (b) Quantitative analysis of the 6E10-positive area (%) in the cortex. (c) Representative Western blot bands of FL-APP, Aβ oligomers and β-actin in cortical and hippocampal lysates. (d–f) Densitometric quantification of FL-APP (d), Aβ oligomers (e, f) relative to β-actin in the cortex and hippocampus. Data are presented as mean ± SEM (n=6 per group). Statistical analyses were performed using two-way ANOVA followed by Tukey’s post-hoc test. *P<0.05, **P<0.01, ***P<0.001 compared with WT.

To evaluate the impact of GPER deficiency on AD-like amyloid pathology and glymphatic function, we performed double-immunofluorescence staining for AQP4 and Aβ (using the 6E10 antibody) in cortical and hippocampal sections of 12-month-old female WT, GPER-KO, and APP/PS1 mice (Fig. 2a). In WT mice, virtually no Aβ plaques were detected (Fig. 2a). Conversely, in GPER-KO and APP/PS1 mice, although the overall immunofluorescence intensity of AQP4 remained clearly visible, its highly restricted perivascular polarization was severely disrupted, manifesting as a diffuse, non-polarized redistribution into the astrocytic soma and parenchymal processes (Fig. 2a). Quantitative analysis of the 6E10-positive area confirmed that the amyloid burden in GPER-KO mice was significantly increased compared to WT controls 4.82% ± 0.51% vs WT: 0.85% ± 0.12%, P<0.01), though it remained lower than that in APP/PS1 mice 6.85% ± 0.62%; Fig. 2b).

To further investigate the biochemical alterations, we performed Western blot analysis on cortical and hippocampal lysates (Fig. 2c). The levels of full-length APP (FL-APP) were exclusively and massively elevated in APP/PS1 mice (Fig. 2d), whereas those in GPER-KO mice remained at baseline levels comparable to WT controls, suggesting that GPER deficiency does not alter APP synthesis. However, GPER-KO mice exhibited a significant increase in neurotoxic Aβ oligomers in both the cortex and hippocampus (Fig. 2e–f), confirming impaired Aβ clearance.

### Astrocyte-specific GPER deletion exacerbates AD-like neuropathology and accelerates cognitive decline in APP/PS1 mice

**Figure 3.**
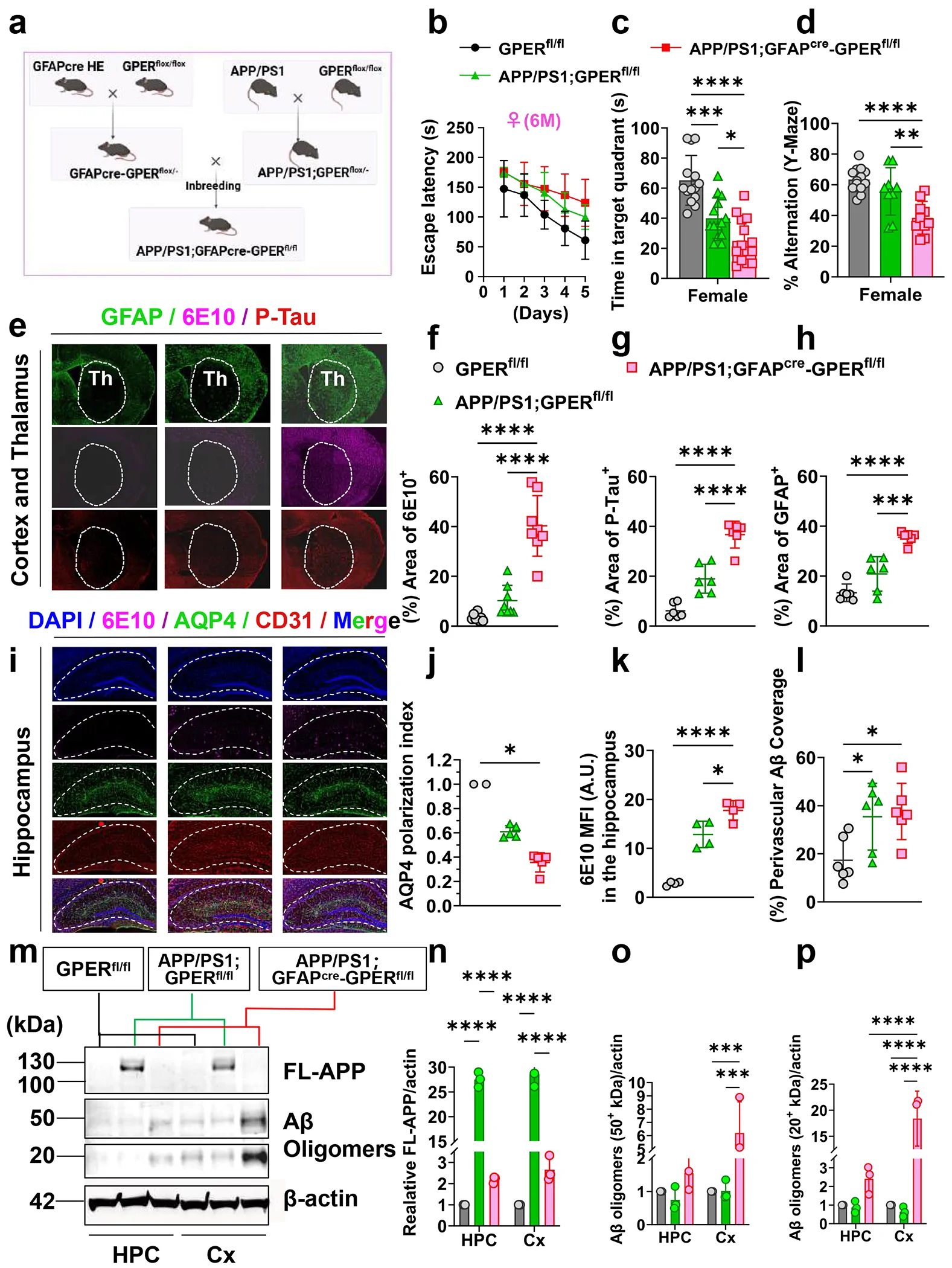
Astrocyte-specific GPER deletion exacerbates AD-like neuropathology and accelerates cognitive decline in APP/PS1 mice. (a) Schematic of the breeding strategy for generating APP/PS1;GFAP^cre^-GPER^fl/fl^ mice. (b-c) Barnes maze performance of 6-month-old (6M^+^) mice, showing escape latency across training days (b) and time spent in the target quadrant during the probe trial (c), indicating significant spatial learning and memory deficits in astrocyte-specific GPER-knockout mice. (d) Spontaneous alternation rates in the Y-maze test, reflecting working memory performance. Data are presented as mean ± SEM (n=9-11 per group). *P<0.05, **P<0.01 vs. GPER^fl/fl(^CTR). (e) Representative whole-brain immunofluorescence images showing GFAP (green), Aβ (6E10, magenta), and P-Tau (red) in the thalamus (Th). Quantitative analysis of the 6E10-positive area (f), P-Tau-positive area (g), and GFAP-positive area (h) in the thalamus. (i) Representative high-resolution confocal images of the hippocampus showing DAPI (blue), Aβ (6E10, magenta), AQP4 (green), and CD31 (red). (j–l) Quantitative analysis of AQP4 perivascular polarization (j), Aβ (6E10) positive area (k), and perivascular Aβ coverage (%, h) in the hippocampus. Data are presented as mean ± SEM(n=6 per group). (m) Representative Western blot bands showing the expression of FL-APP, Aβ oligomers and β-actin (loading control) in the cortex and hippocampus. (n–p) Densitometric quantification of FL-APP (n), Aβ oligomers (o, p) relative to β-actin in the cortex and hippocampus. Statistical significance was determined by two-way ANOVA followed by Tukey’s post-hoc test.

To assess the impact of astrocytic GPER deficiency on cognitive function in early-stage Alzheimer’s disease^[27]^, we also performed Barnes maze and Y-maze tests on 6-month-old female mice (Fig. 3a) from three genotypes: GPER^fl/fl^ (CTR, n=11), APP/PS1-GPER^fl/fl^ (n=9, Fig. S1a), and APP/PS1;GFAP^cre^-GPER^fl/fl^ (n=10, Fig. S1b). In the Barnes maze, APP/PS1-GPER^fl/fl^ mice exhibited significantly longer escape latencies and less time in target quadrant compared to GPER^fl/fl^ controls throughout the training days, indicating impaired spatial learning (Fig. 3b–c). Notably, APP/PS1;GFAP^cre^-GPER^fl/fl^ mice showed the most pronounced deficits, with escape latencies consistently higher than both APP/PS1-GPER^fl/fl^ and GPER^fl/fl^ groups, suggesting that astrocyte-specific GPER knockout further exacerbates spatial memory impairment in the APP/PS1 background (Fig. 3b–c). In the Y-maze test, spontaneous alternation rates were significantly reduced in APP/PS1-GPER^fl/fl^ mice compared to controls, reflecting compromised working memory (Fig. 3d). APP/PS1;GFAP^cre^-GPER^fl/fl^ mice displayed the lowest alternation rates among all groups, confirming that loss of astrocytic GPER aggravates cognitive dysfunction in the context of amyloid pathology.

Whole-brain immunofluorescence staining revealed that these mice exhibited widespread Aβ (6E10) accumulation and robust P-Tau expression, particularly in the thalamic (Th) regions, compared to their littermate controls (Fig. 3e–h). High-resolution imaging of the hippocampus further demonstrated that astrocytic GPER deficiency led to a significant loss of perivascular AQP4 polarization, which was accompanied by extensive Aβ deposition and vascular disruption, as evidenced by CD31 staining (Fig. 3i–l). Biochemical analyses of cortical and hippocampal lysates confirmed these findings (Fig. 3m); while FL-APP levels remained consistent with the APP/PS1 transgene status, astrocyte-specific GPER-deficient mice exhibited a marked increase in neurotoxic Aβ oligomers (Fig. 3n–p), demonstrating that the loss of astrocytic GPER triggers Aβ oligomerization and accelerates amyloid-associated neuropathology. Collectively, these results demonstrate that astrocytic GPER is essential for maintaining cognitive function, and its deficiency significantly accelerates cognitive decline in the context of amyloid pathology.

### GPER deficiency impairs neurovascular coupling and hemodynamic responses in aged mice

**Figure 4.**
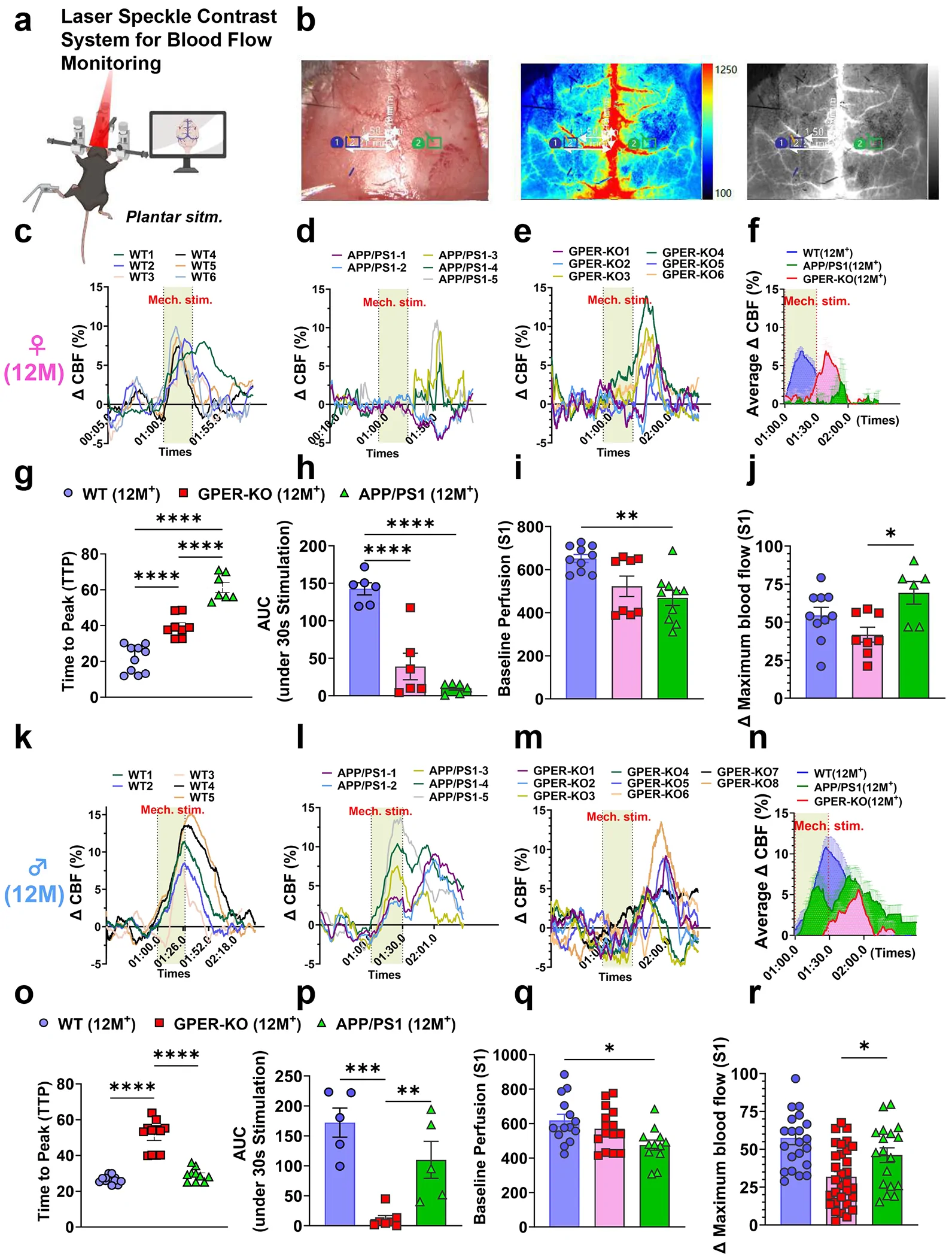
GPER deficiency impairs neurovascular coupling dynamics and limits vascular reserve in the somatosensory cortex of aged mice. (a, b) Experimental paradigm and representative LSCI imaging. Schematic illustration of the setup and 30-s hindpaw stimulation timeline (a), with the Region of Interest (ROI) defined over the contralateral somatosensory cortex (b). Representative pseudocolor maps visualize cortical perfusion distribution at baseline and peak response across WT, GPER-KO, and APP/PS1 mice, where warmer colors indicate higher blood flow intensity. (c-e, k-m) Temporal profiles of the hemodynamic response, expressed as relative cerebral blood flow change ΔCBF/CBF_baseline_). The response curves in GPER-KO and APP/PS1 mice are notably blunted and delayed compared to the robust, rapid response observed in WT mice. (f–j, n-r) Quantitative analysis of hemodynamic response characteristics: Peak amplitude (f, n), Time-to-Peak (g, o) and Area Under the Curve (AUC) during the 30-s stimulation (h, p). GPER-KO mice exhibited a significantly reduced response amplitude and delayed kinetics. Quantitative analysis of absolute baseline cerebral blood flow (i, q). While basal perfusion was preserved in GPER-KO mice (comparable to WT), in contrast to the reduced baseline observed in APP/PS1 mice, the maximal evoked blood flow in GPER-KO mice was severely compromised (j, r). Data are presented as mean ± SEM (n=9-12 per group). *p<0.05, **p<0.01,***p<0.001 vs. WT.

Given that GPER is distinctively expressed in key components of NVU^[11]^, we attempted to dissect the critical role of GPER in maintaining the functional integrity of NVU by employing Laser Speckle Contrast Imaging (LSCI, RWD, Shenzhen, China) to systematically evaluate cerebral blood flow (CBF) dynamics^[28]^ in the somatosensory cortex of 12-month-old WT, GPER-KO and APP/PS1 transgenic mice. This investigation is prompted by evidence that GPER mediates rapid non-genomic vasodilation and regulates vascular tone through the nitric oxide (NO) signaling pathway^[29, 30]^. Based on this, we established a paradigm of somatosensory stimulation-evoked cerebral blood flow (CBF) response, in which a constant mechanical pressure of 33 N was applied to the hindpaw of the mice to assess their neurovascular coupling (NVC) response (Fig. 4a). Upon a 30-second stimulation, WT mice exhibited a robust, localized surge in CBF within the contralateral somatosensory cortex, a hallmark of physiological functional hyperemia (Fig. 4b). In contrast, both GPER-KO and APP/PS1 mice exhibited a markedly blunted hemodynamic response. Quantitative analysis of the time-intensity curves revealed distinct hemodynamic profiles among genotypes in both female (Fig. 4c–j) and male (Fig. 4k–r) cohorts.

In WT mice, we observed a rapid attainment of peak blood flow upon stimulation onset followed by a sustained high-level plateau, reflecting physiological functional hyperemia (Fig. 4c, k). In contrast, the hemodynamic response curves of the GPER-KO group exhibited a consistent trend of delay and attenuation (Fig. 4e, m), mirroring the deficits observed in APP/PS1 mice (Fig. 4d, l). Specifically, GPER deficiency resulted in a substantial reduction in both peak amplitude and the Area Under the Curve (AUC) (Fig. 4h, p; female: P<0.0003), indicating a net loss of total perfusion volume during neuronal activation. The rapid vasodilation observed in WT controls was replaced by a sluggish response in GPER-KO mice, evidenced by a significantly prolonged time-to-peak (Fig. 4g, o). Further quantification of absolute blood flow parameters highlighted a striking dissociation between resting and stimulated states across genotypes. Regarding baseline perfusion (Fig. 4i, q), the APP/PS1 group exhibited significantly lower values compared to controls; conversely, GPER-KO mice maintained basal flow levels indistinguishable from WT mice. However, the magnitude of the stimulation-evoked hemodynamic deficits in GPER-KO mice was comparable to that of the APP/PS1 group. This suggests that while GPER is dispensable for maintaining basal perfusion, it is critical for mobilizing the vascular reserve during neuronal activation. Collectively, these findings indicate that GPER loss is sufficient to precipitate severe vascular dysfunction—characterized by a failure in neurovascular coupling—akin to the pathological states observed in Alzheimer’s disease models, a phenotype that remains consistent across both sexes. Interestingly, while GPER-KO mice displayed delayed response kinetics, their maximal CBF change in the S1 cortex remained comparable to that of WT controls (Fig. 4j, r). This finding suggests that GPER deficiency does not impair the absolute maximal vasodilatory capacity of the cerebral vasculature; rather, it disrupts the temporal efficiency and sustained mobilization of vascular reserves during neuronal activation.

### GPER deficiency impairs capillary-level hemodynamic responses in 12-month-old mice

To further elucidate the cellular mechanisms underlying NVC impairment, we performed in vivo two-photon imaging to monitor RBC velocity in individual cortical capillaries of 12-month-old female WT and GPER-KO mice during hindpaw electrical stimulation[31]. To quantify capillary-level hemodynamics, line-scan imaging was performed along the longitudinal axis of individual capillaries. This technique generated space-time images where the x-axis represents spatial position along the capillary and the y-axis represents time. In these images, red blood cells (RBCs) appeared as dark, elongated shadows against the fluorescently labeled blood plasma (FITC-dextran). The velocity of RBCs (V*RBC*) was determined by the slope of these dark streaks in the space-time images[32]. Specifically, the angle (θ) of the streaks relative to the time axis is inversely proportional to the velocity, calculated according to the formula: V*RBC*=[1/tan(θ)]×pixel size/sampling interval. To ensure accuracy, velocity data were processed using a custom MATLAB algorithm (LS-PIV)[21]. Initially, space-time images were subjected to high-pass filtering to enhance the contrast of the RBC shadows. The velocity was then extracted by calculating the cross-correlation of successive lines within the space-time image, allowing for the precise tracking of RBC displacement over time. Finally, the raw velocity traces were smoothed using a 1-s boxcar filter in Igor Pro to eliminate cardiac and respiratory artifacts, yielding high-fidelity hemodynamic waveforms for subsequent statistical analysis[33].

**Figure 5.**
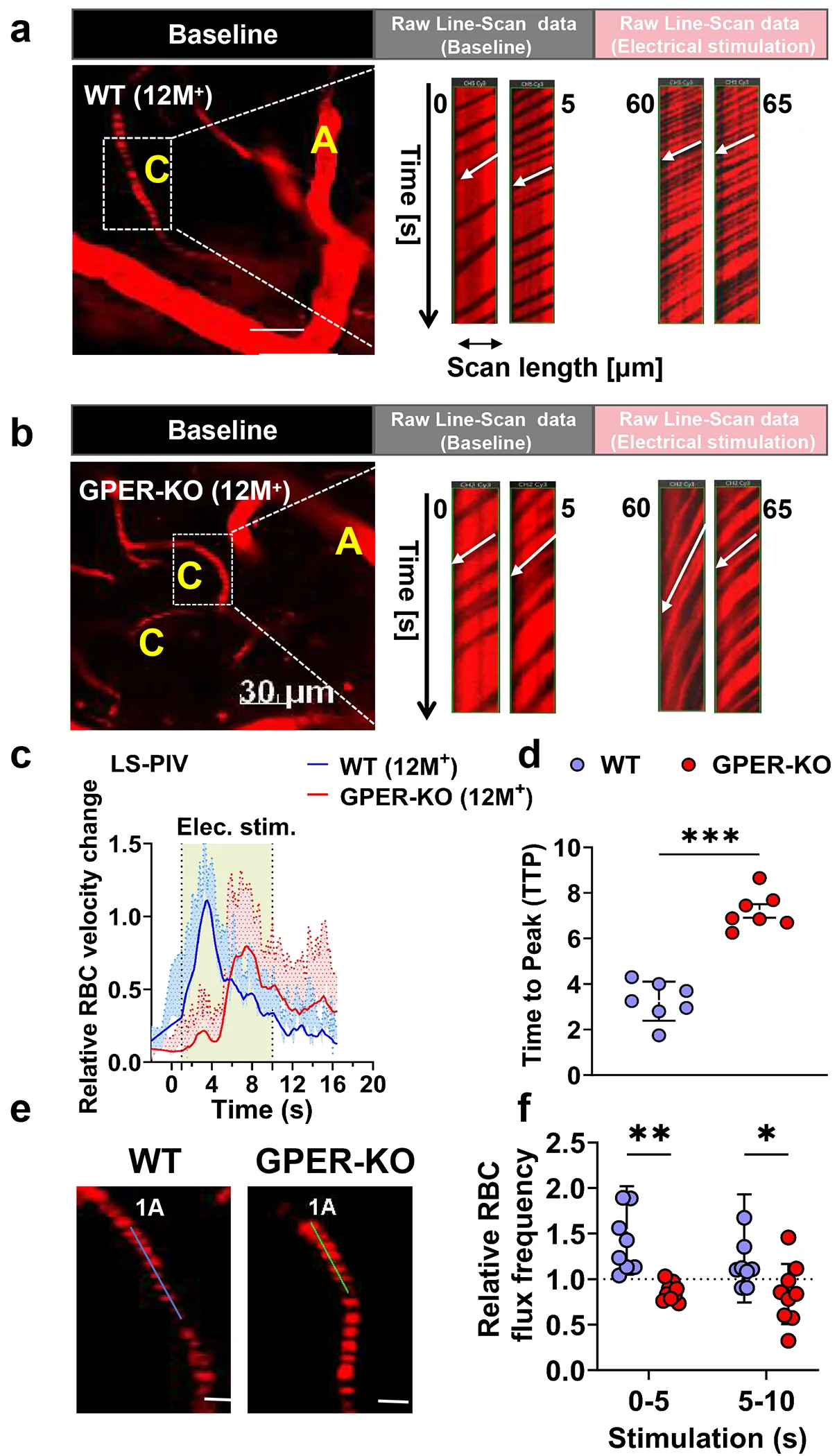
GPER deficiency impairs capillary-level hemodynamic responses in 12-month-old mice. (a,b) Representative space-time images of RBC flow in cortical capillaries of 12-month-old female WT (a) and GPER-KO (b) mice at baseline and during electrical hindlimb stimulation. (c) Hemodynamic waveforms showing real-time changes in RBC velocity. (d) Quantification of the time-to-peak of RBC velocity response. (e) Peak RBC velocity change. (f) Comparison of RBC velocity change between the first 5 s and the latter 5 s of the 10-s stimulation period. Data are mean ± SEM (n=6–8 capillaries from 5 mice per group). *P<0.05, **P<0.05 vs. WT.

In WT mice, electrical stimulation of the hindpaw induced a rapid and significant increase in RBC velocity, reflecting efficient capillary-level functional hyperemia (Fig. 5a). In striking contrast, 12-month-old female GPER-KO mice exhibited a significantly attenuated velocity response (Fig. 5b). Quantitative analysis of the space-time images revealed that while the basal RBC velocity was comparable between genotypes, the stimulation-induced velocity surge was markedly blunted in GPER-KO mice (Fig. 5c). Specifically, both the peak RBC velocity and the total velocity change during stimulation were significantly reduced in the GPER-KO group (Fig. 5d–f). These microvascular findings confirm that GPER deficiency in aged mice disrupts the ability of capillaries to dynamically adjust blood flow in response to neuronal activity. This loss of capillary-level “on-demand” perfusion suggests that GPER is a crucial regulator of the micro-hemodynamic responses required for metabolic support during neuronal activation in the aging brain.

### Transcriptomic analysis revealed that GPER activation in pericytes reinforces the *Lrp1*-dependent endocytic pathway to drive Aβ clearance

**Figure 6.**
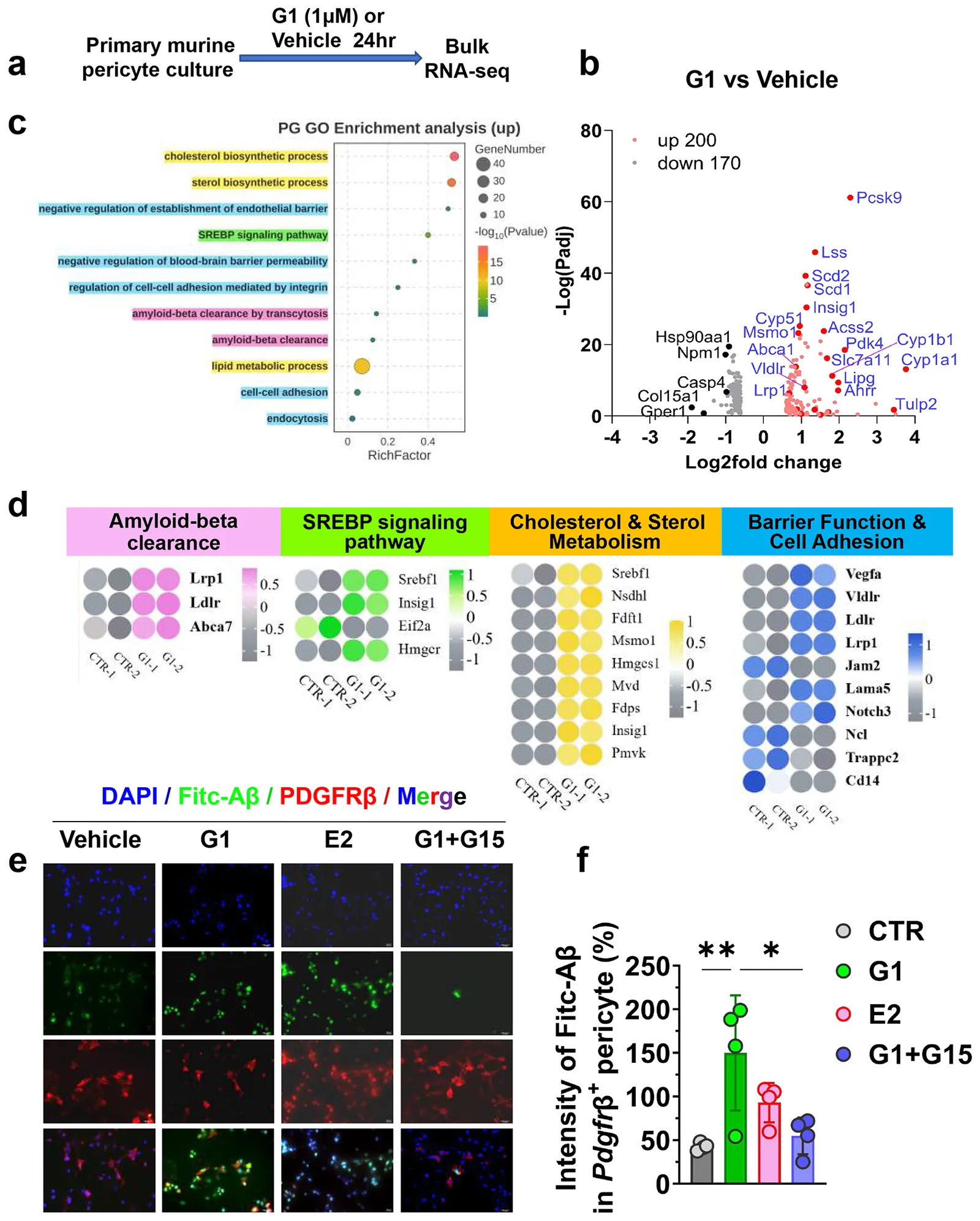
GPER activation drives *Lrp1*-dependent Aβ clearance in pericytes. **(a)** Schematic of experimental design. (b) Volcano plot highlighting differentially expressed genes. (c) GO enrichment analysis showing upregulated pathways following G1 treatment. (d) Heatmap of functional gene clusters related to Aβ clearance, SREBP signaling, cholesterol metabolism, and barrier function. (e, f) Quantification of FITC-Aβ phagocytosis in pericytes treated with or without G1. Data are mean ± SEM. *p < 0.05, **p < 0.01, and ***p < 0.001.

To elucidate the molecular mechanisms by which GPER activation in murine pericytes influences Aβ clearance, we performed transcriptomic profiling following G1 (GPER agonist) treatment of cultured primary pericytes (Fig. 6a). Volcano plot and Gene Ontology (GO) enrichment analysis indicated coordinated upregulation of key genes involved in pericyte functionality (Fig. 6b–c). Specifically, the differentially expressed genes (DEGs) consisted of four functional clusters: Aβ clearance, SREBP signaling, cholesterol/sterol metabolism, and barrier function/cell adhesion (Fig. 6d). G1 treatment markedly upregulated key receptors involved in Aβ uptake, such as *Lrp1*, *Ldlr*, and *Abca7*. Concurrently, the SREBP signaling pathway—a master regulator of lipid homeostasis—was activated, as evidenced by the elevated expression of *Srebf1*, *Insig1*, and *Hmgcr*. Notably, genes critical for barrier integrity and structural support, including *Vegfa*, *Jam2*, and *Notch3*, were also upregulated, suggesting that GPER signaling optimizes the neurovascular unit (NVU) microenvironment.

To substantiate these transcriptomic findings, we assessed the functional impact of GPER activation on pericyte-mediated Aβ phagocytosis. Functional FITC-Aβ uptake assays revealed that G1-treated pericytes exhibited a significantly higher phagocytic index compared to control cells (Fig. 6e–f). These data establish a mechanistic nexus wherein GPER signaling drives a synergistic program that integrates structural reinforcement with lipid homeostasis to facilitate efficient *Lrp1*-dependent endocytic Aβ clearance in pericytes.

### Transcriptome analysis reveals that loss of GPER compromises astrocytic gap junction connectivity and *AQP4*-mediated glymphatic clearance

**Figure 7.**
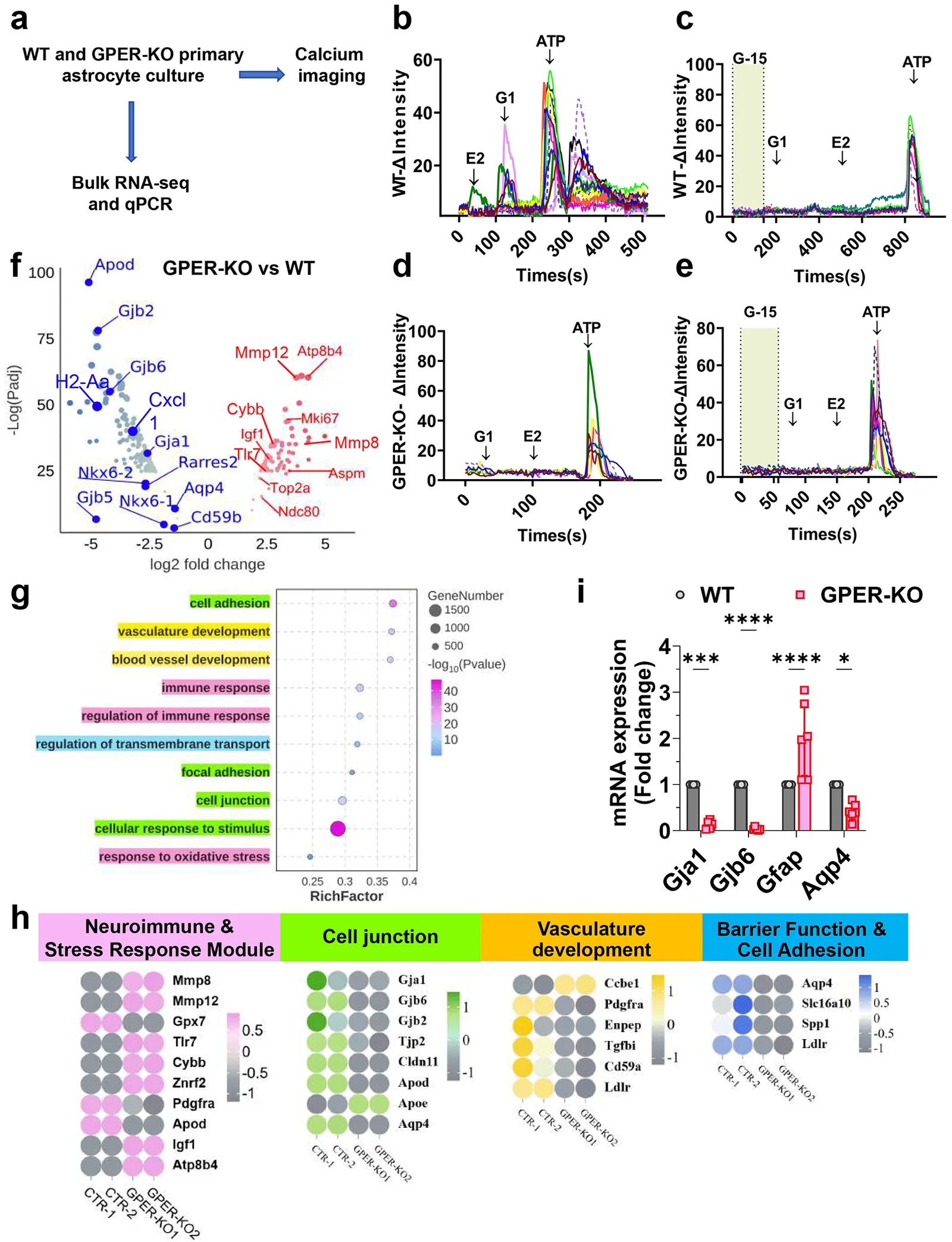
Transcriptome analysis reveals that loss of GPER knockout compromises astrocytic gap junction connectivity and *AQP4*-mediated glymphatic clearance. (a) Schematic illustration of the experimental design for primary astrocyte isolation, RNA sequencing, and calcium imaging. (b-e) Representative traces of Fluo-4 calcium imaging. In WT astrocytes, application of the GPER agonist G1 or estradiol (E2) induces intracellular calcium transients, which are further potentiated by subsequent ATP stimulation (b). In contrast, these calcium responses are completely abolished in GPER-KO astrocytes (d). WT astrocytes pretreated with the GPER antagonist G15 (1 μM) failed to respond to G1 or E2. However, subsequent application of ATP (100 μM) elicited robust calcium transients, confirming cell viability and the efficacy of the pharmacological blockade (c). Similarly, astrocytes isolated from GPER-KO mice exhibited no calcium response upon stimulation with G1 or E2, but maintained intact, robust calcium transients in response to ATP (e). (f) Volcano plot illustrating the global transcriptomic shift in GPER-KO astrocytes compared to WT. Key downregulated genes essential for structural integrity and intercellular communication (*AQP4*, *Gja1*, *Gjb6*, *Gjb2*) are highlighted in blue, while upregulated inflammatory and tissue-remodeling genes (*Mmp12*, *Cybb*, *Tlr7*, *Atp8b4*) are highlighted in red. (g) GO enrichment analysis of differentially expressed genes in GPER-KO versus WT primary astrocytes, highlighting significant alterations in cell junction, regulation of transmembrane transport, immune response, and vasculature development. (h) Modular heatmap categorizing differentially expressed genes into functional clusters: Neuroimmune & Stress Response, Cell junction, Vasculature development, and Barrier Function & Cell Adhesion. (i) Quantitative RT-PCR validation of selected up- and downregulated genes identified by RNA-seq. Data are mean ± SEM. **P<0.01, ***P<0.001 vs. WT (CTR).

Having established that GPER signaling potentiates pericyte-mediated Aβ phagocytosis, we next interrogated whether GPER exerts a parallel regulatory influence on astrocytes, the other critical component of the neurovascular unit (NVU). Astrocytes maintain NVU homeostasis through endfoot processes that regulate blood flow and facilitate solute clearance via the aquaporin-4 (*AQP4*)-dependent glymphatic pathway^[34]^. To delineate the transcriptional landscape governed by GPER, we performed RNA sequencing and calcium imaging on primary astrocytes isolated from WT and GPER-KO mice (Fig. 7a). GPER activation by G1 or estradiol (E2) triggered pronounced calcium responses in a proportion (64/201, approximately 31.84%) of WT astrocytes (Fig. 7b). Pretreatment with the GPER antagonist G15 abolished G1- and E2-induced calcium responses in WT astrocytes without affecting subsequent ATP-evoked transients (Fig. 7c). Astrocytes from GPER-KO mice exhibited robust calcium transients to ATP but failed to respond to either G1 or E2 (Fig. 7d–e). These results confirmed expression of functional GPER in a population of astrocytes, consistent with its role in modulating neurovascular coupling and gliotransmission^[35]^.

Differential expression analysis revealed a profound transcriptomic shift in GPER-KO astrocytes. Volcano plot analysis highlighted a striking suppression of genes essential for astrocytic structural integrity and intercellular communication, including *AQP4*, *Gja1* (Connexin 43), *Gjb6* (Connexin 30), and *Gjb2* (Fig. 7f). Conversely, we observed the upregulation of genes such as *Atp8b4* and *Mki67*, alongside key inflammatory and tissue-remodeling markers including *Tlr7*, *Cybb*, *Mmp12*, and *Mmp8* (Fig. 7f), suggesting that loss of GPER drives astrocytes to polarize toward a neurotoxic, pro-inflammatory (A1-like) or immune-activated state^[36]^. Consistent with these global transcriptomic alterations, GO enrichment analysis identified “cell junction,” “regulation of transmembrane transport,” “vasculature development,” and “immune response” as the most significantly altered pathways (Fig. 7g). To systematically categorize these changes, we constructed a modular heatmap, which confirmed a coordinated collapse in the expression of gene clusters associated with “Cell junction” and “Barrier Function & Cell Adhesion” in GPER-deficient astrocytes (Fig. 7h). To validate these transcriptomic findings, we performed quantitative RT-PCR. Consistent with the RNA-seq data, GPER-KO astrocytes exhibited a marked reduction in the mRNA levels of *AQP4*, *Gja1*, *Gjb6*, *Gjb2* and *Cldn11* (Fig. 7i). Since AQP4 polarization at perivascular endfeet is rate-limiting for glymphatic exchange^[37, 38]^, and connexins are pivotal for maintaining the astrocytic syncytium^[39]^, this downregulation suggests that GPER loss fundamentally impairs the brain’s waste clearance machinery.

### GPER knockout impairs glymphatic clearance in the aged cortex

**Figure 8.**
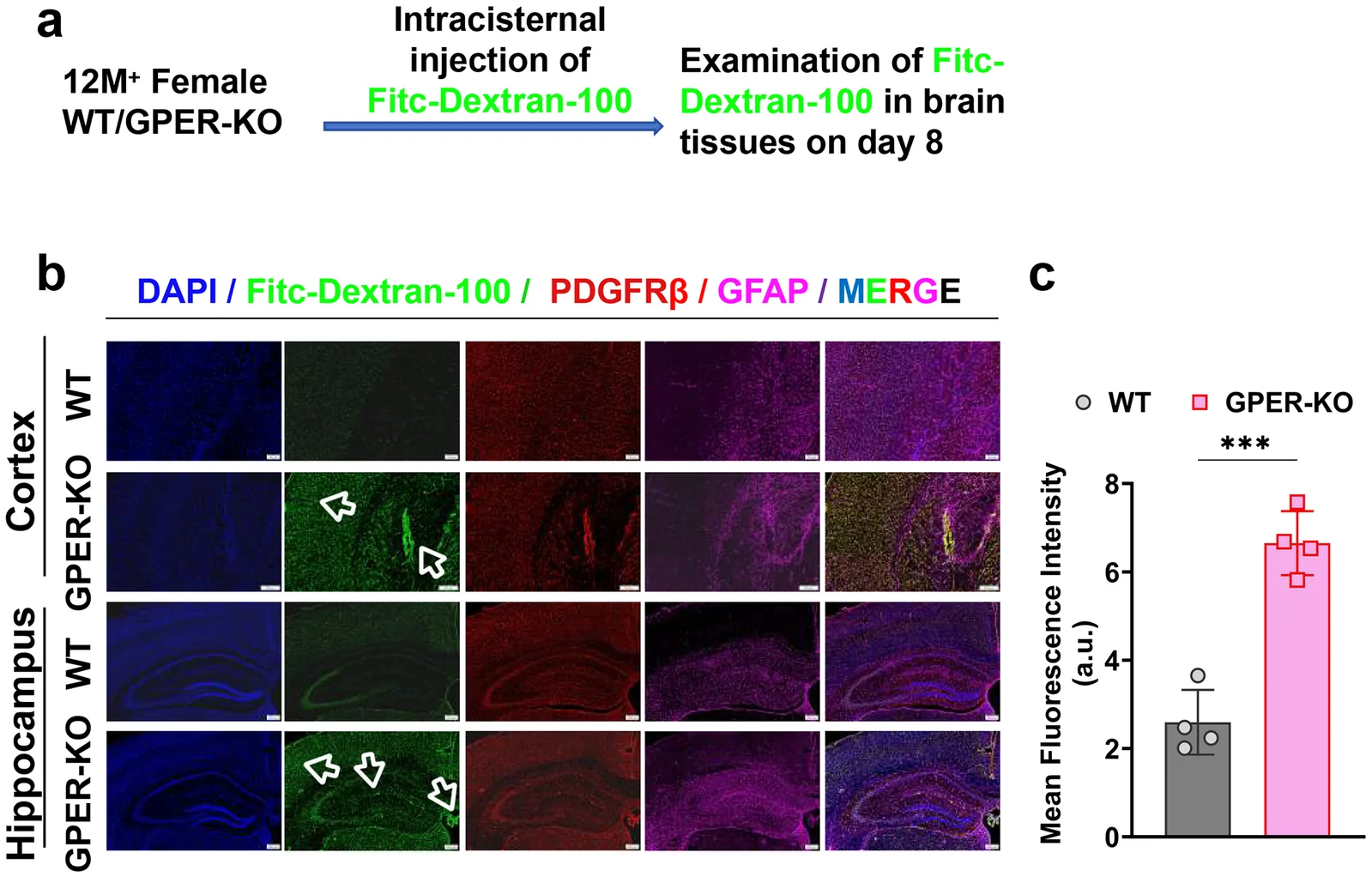
GPER deficiency impairs glymphatic clearance and disrupts astrocytic barrier function. (a) Schematic of the Fitc-dextran-100 glymphatic clearance assay. (b) Representative immunofluorescence images of Fitc-dextran (green), PDGFRβ (red), and GFAP (magenta) in the cortex and hippocampus. Scale bars: 100 μm. (c) Quantification of Fitc-dextran retention in WT and GPER-KO mice. Data are mean ± SEM (n=6 mice per group). **P<0.01 vs. WT.

To elucidate the functional consequences of GPER deficiency on solute clearance, we performed cisterna magna injections of Fitc-dextran-100 in 12-month-old female WT and GPER-KO mice (Fig. 8a). Immunofluorescence staining for PDGFRβ (red) and GFAP (magenta) revealed the spatial distribution of the tracer (green) within the brain parenchyma on day 8 after injection. In WT mice, Fitc-dextran was efficiently cleared from the brain, with minimal residual signal observed in both perivascular and parenchymal regions (Fig. 8b). In contrast, GPER-KO mice exhibited pronounced retention of Fitc-dextran, as indicated by strong green fluorescence accumulation within the cortex and hippocampus (Fig. 8b). Quantitative analysis confirmed a significant increase in Fitc-dextran retention in GPER-KO mice compared to WT controls (Fig. 8c), demonstrating that GPER is essential for efficient glymphatic clearance in the aged brain.

### GPER might interact with Aβ and its deficiency drives Aβ accumulation at the gliovascular interface

**Figure 9.**
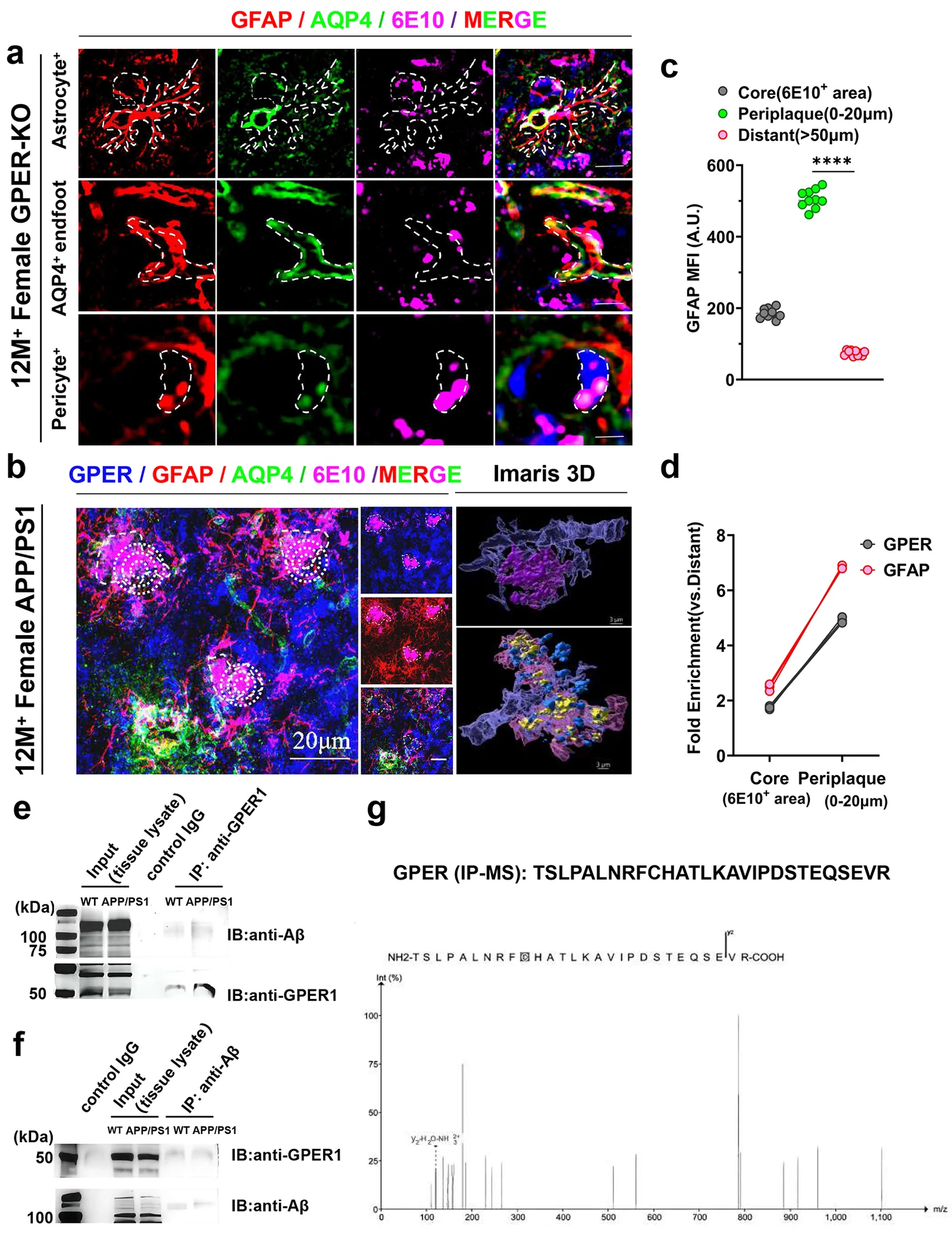
GPER might interact with Aβ and its deficiency drives Aβ accumulation at the gliovascular interface. (a) Representative high-resolution multiplex immunofluorescence images of the cortex from aged GPER-KO mice. The images show the spatial distribution of GFAP (red, astrocytes), AQP4 (green, astrocytic endfeet), and Aβ (6E10, magenta). Dashed outlines highlight the striking colocalization and sequestration of Aβ aggregates within AQP4^+^ astrocytic endfeet domains at the perivascular niche. Dashed lines indicate regions of colocalization. Scale bars: 10 μm. (b-d) Representative multiplex immunofluorescence images of the cortex from 12-month-old APP/PS1 mice, demonstrating the spatial colocalization of GPER (blue), GFAP (red), AQP4 (green), and Aβ (6E10, magenta). Dotted circles indicate regions of intense GPER and Aβ overlap within astrocytic domains. Scale bar: 20 μm. Imaris 3D reconstruction of the gliovascular interface from APP/PS1 mice, providing visual evidence of the intimate spatial proximity and potential physical interaction between GPER (blue) and Aβ aggregates (magenta/yellow) (b), Quantitative analysis of relative GFAP (grey) and GPER (red) fluorescence intensities in regions distant from versus proximal to Aβ plaques (corresponding to the dashed areas in Figure 8b). The data demonstrate a robust and concurrent enrichment of both reactive astrocytes (GFAP) and GPER in the immediate vicinity of amyloid deposits (c-d). Each connected line represents paired distant and proximal measurements from individual plaques. (e-f) Co-immunoprecipitation (Co-IP) analysis of brain lysates. Immunoblotting demonstrates the formation of a stable GPER–Aβ complex in vivo, supporting the potential physical interaction between GPER and Aβ. (g) Representative mass spectrum from Immunoprecipitation-Mass Spectrometry (IP-MS) analysis. The spectrum identifies specific GPER peptide fragments (sequence shown) within Aβ immunoprecipitates, suggesting a direct molecular interaction.

To investigate whether the observed impairment in glymphatic clearance stems from a direct molecular interplay between GPER and amyloid pathology, we performed high-resolution multiplex immunofluorescence on cortical sections. In aged GPER-KO mice, Aβ aggregates (6E10, magenta) exhibited striking colocalization with GFAP^+^ astrocytic processes and were sequestered within AQP4^+^ endfeet domains (Fig. 9a). Spatial analysis confirmed that Aβ deposition preferentially occurred in perivascular niches and pericyte-adjacent regions (Fig. 9a).

To elucidate the molecular relationship between GPER and Aβ, we performed multiplex immunofluorescence in APP/PS1 mice, revealing that GPER (blue) and Aβ (magenta) are highly colocalized within astrocytic domains and perivascular regions (Fig. 9b–d). Imaris 3D reconstruction provided visual evidence of this intimate spatial proximity between GPER and Aβ aggregates (Fig. 9b). To validate the hypothesis of a direct physical interaction, we conducted co-immunoprecipitation (Co-IP) and IP-MS analysis. Co-IP confirmed the formation of a stable GPER-Aβ complex in brain lysates (Fig. 9e–f), while IP-MS identified specific Aβ peptide fragments within GPER immunoprecipitates, as evidenced by the representative mass spectrum (Fig. 9g). These findings demonstrate that GPER directly interacts with Aβ, suggesting a possible role for GPER in recognizing and sequestering Aβ at the neurovascular interface to facilitate its clearance.

### GPER Maintains Neurovascular Unit Ultrastructure and Orchestrates Pericyte-Astrocyte Coupling for Efficient Aβ Clearance

**Figure 10.**
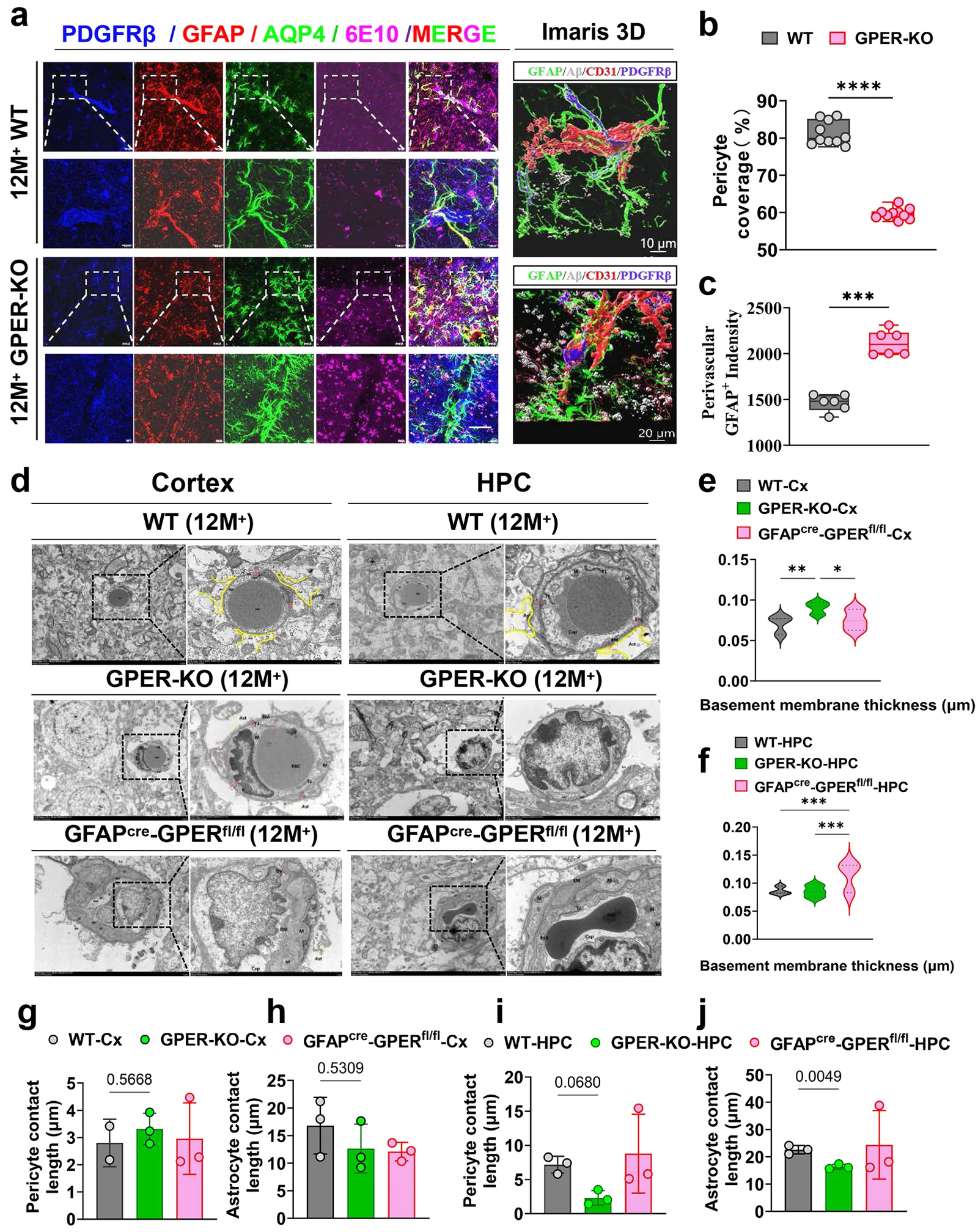
GPER maintains neurovascular unit ultrastructure and pericyte-astrocyte interactions. (a) Representative multiplex immunofluorescence images showing PDGFRβ (blue), GFAP (red), AQP4 (green), and Aβ (6E10, magenta) in the cortex of 12-month-old WT and GPER-KO mice. Imaris 3D reconstruction of the neurovascular niche, illustrating the spatial relationship between astrocytes (green), vessels (CD31, red), pericytes (blue), and Aβ (white/grey). (b, c) Quantitative analysis of pericyte coverage (b) and relative perivascular GFAP^+^ intensity (c). (d) Representative transmission electron microscopy (TEM) images showing the ultrastructure of the vascular basement membrane (BM) and astrocytic endfeet across WT, GFAP^cre^-GPER^fl/fl^, and GPER-KO mice. (e-f) Quantitative analysis of BM thickness and BM heterogeneity based on TEM images. (g, h) Quantitative analysis of the contact length (μm) between pericytes and astrocytes in the cortex of WT, GFAP^cre^-GPER^fl/fl^, and GPER-KO mice. (i, j) Corresponding quantification of the pericyte-astrocyte contact length (μm) in the hippocampus. Data are presented as mean ± SEM. Grey = WT, Green = GFAP^cre^-GPER^fl/fl^, Pink = GPER-KO.

To investigate the relationship between GPER deficiency and blood-brain barrier (BBB) integrity^[40]^, we performed multiplex immunofluorescence staining on cortical sections from 12-month-old female WT and GPER-KO mice. In WT mice, PDGFRβ^+^ pericytes (blue), GFAP^+^ astrocytes (red), and AQP4^+^ endfeet (green) formed a highly organized, dense network with minimal Aβ (6E10, magenta) deposition (Fig. 10a). Conversely, GPER-KO mice exhibited a disorganized neurovascular niche, characterized by a significant reduction in pericyte coverage and massive Aβ accumulation along the vascular interface (Fig. 10b–c). Imaris 3D reconstruction further visualized this severe disruption, highlighting the breakdown of pericyte-astrocyte coupling and the perivascular sequestration of Aβ aggregates in GPER-deficient brains. These findings indicate that GPER is essential for maintaining BBB integrity and preventing vascular amyloid deposition during aging.

Then we want to elucidate the ultrastructural consequences of GPER deficiency on the neurovascular unit^[41]^, we utilized transmission electron microscopy (TEM) across 12-month-old WT, astrocyte-specific GPER knockout (GFAP^cre^-GPER^fl/fl^), and global GPER-KO mice. In WT mice, the vascular basement membrane (BM) was thin, continuous, and well-organized. In contrast, both GFAP^cre^-GPER^fl/fl^ and global GPER-KO mice displayed marked BM thickening, fragmentation, and increased electron density, accompanied by swollen astrocytic endfeet (Fig. 10d). Quantitative analysis confirmed a significant increase in BM thickness and heterogeneity in both GPER-deficient models compared to WT controls, indicating that astrocytic GPER is indispensable for maintaining basement membrane homeostasis (Fig. 10e–f). Both GPER-KO and GFAP^cre^-GPER^fl/fl^ mice exhibited marked thickening, fragmentation, and irregularity of the basement membrane, accompanied by increased electron density and disrupted association with adjacent endothelial and astrocytic structures (Fig. 10g–j). These changes were accompanied by increased electron density and heterogeneity of the basement membrane, further supporting the notion of neurovascular unit remodeling. Collectively, these data demonstrate that GPER is essential for maintaining basement membrane homeostasis, pericyte-astrocyte interactions, and neurovascular integrity in the aging brain^[42, 43]^.

## Discussion

The neurovascular unit (NVU) represents a complex functional module where vascular cells and glia coordinate to match neuronal energy demand with blood flow and to clear metabolic waste. In this study, we identify the G protein-coupled estrogen receptor (GPER) as a critical regulator of NVU homeostasis, specifically orchestrating the synergistic functions of astrocytes and pericytes to maintain NVC and drive Aβ clearance. Our findings demonstrate that GPER deficiency precipitates a cascade of neurovascular dysfunctions—ranging from impaired hemodynamic responses and basement membrane thickening to the collapse of glymphatic transport—that collectively accelerate cognitive decline and amyloid pathology in an age- and sex-dependent manner.

A central finding of our work is the critical role of GPER in maintaining the “vascular reserve” required for NVC. While macroscopic basal perfusion remained intact in GPER-KO mice, the hemodynamic response to somatosensory stimulation was severely blunted, mirroring the deficits observed in APP/PS1 mice. Crucially, this macroscopic impairment was corroborated at the microscopic level by our in vivo two-photon imaging. At the single-vessel resolution, GPER-deficient mice exhibited a profound failure in stimulus-evoked vasodilation of penetrating arterioles and capillaries. This microvascular rigidity provides direct evidence that GPER signaling is indispensable for the rapid, dynamic adjustment of vessel caliber mediated by the gliovascular interface^[16]^. Mechanistically, the downregulation of connexins (*Gja1*,*Gjb2*, *Gjb6*, etc) in GPER-KO astrocytes indicates a breakdown of the astrocytic syncytium, which is required for the spatial buffering of ions and propagation of vasoactive signals^[44]^.

Beyond blood flow regulation, our study uncovers a novel mechanism by which GPER drives Aβ clearance through pericytes. Transcriptomic profiling revealed that GPER activation reprograms pericytes towards a pro-clearance phenotype by upregulating *Lrp1* and activating the SREBP-dependent lipid metabolic pathway. This establishes a “metabolic-proteostatic axis” where GPER not only enhances the scavenger receptor capacity but also modulates membrane lipid composition to facilitate endocytosis^[45]^. The observation that GPER deficiency leads to Aβ accumulation specifically at pericyte-deficient vascular niches further underscores the protective role of GPER-mediated pericyte coverage. This aligns with recent evidence suggesting that pericyte loss is a primary driver of BBB breakdown and amyloid accumulation in AD^[46]^.

Crucially, we provide ultrastructural and molecular evidence that GPER acts as a structural determinant of the glymphatic system. The polarization of AQP4 at astrocytic endfeet is the rate-limiting step for CSF-ISF exchange^[34]^. We found that GPER deficiency causes a profound loss of perivascular AQP4 polarization, accompanied by basement membrane thickening and fragmentation. This structural remodeling creates a physical barrier to solute transport, trapping Aβ within the perivascular spaces and brain parenchyma. Most intriguingly, our discovery of a direct physical interaction between GPER and Aβ suggests that GPER may function as a membrane-bound “scaffold” or co-receptor that facilitates the recognition or anchoring of Aβ for clearance processes. The loss of this interaction in GPER-KO mice likely contributes to the sequestration of Aβ aggregates at the gliovascular interface.

In conclusion, we propose a unified mechanistic model wherein GPER serves as a linchpin of neurovascular health and Aβ clearance[47]. Under physiological conditions, GPER is highly enriched in astrocytic endfeet and pericytes, sustaining the structural and functional integrity of the neurovascular unit (NVU). It promotes AQP4 polarization, maintains gap and tight junction protein expression (e.g., *Cx43*, *Gjb2*, *Gjb6*), and preserves pericyte coverage, thereby ensuring robust blood-brain barrier (BBB) function and efficient glymphatic clearance. Conversely, GPER deficiency triggers a cascade of NVU dysfunction, characterized by the loss of AQP4 polarity, downregulation of junctional proteins, and pericyte detachment. This disruption leads to BBB breakdown, impaired neurovascular coupling (NVC), and the subsequent accumulation of Aβ within perivascular and parenchymal compartments.

Importantly, the sexual dimorphism observed in our cognitive and pathological assessments underscores the clinical relevance of GPER. The more severe phenotype in GPER-deficient females parallels the higher prevalence of AD in women and the accelerated cognitive decline following post-menopausal estrogen loss. Ultimately, the loss of GPER initiates a vicious cycle of vascular uncoupling and amyloid aggregation. By demonstrating that GPER activation can restore astrocytic syncytium and enhance pericyte phagocytosis, our findings suggest that targeting GPER offers a promising strategy to rejuvenate the aging NVU (Graphical Abstract). Specific GPER agonists may thus provide profound neurovascular protection without the systemic side effects associated with classical estrogen therapy.

## Conclusion

The present study identifies GPER as a critical regulator of neurovascular unit homeostasis, orchestrating the synergistic functions of astrocytes and pericytes to maintain neurovascular coupling and facilitate efficient Aβ clearance. Our findings demonstrate that GPER deficiency precipitates a cascade of neurovascular dysfunctions–ranging from impaired hemodynamic responses and basement membrane remodeling to the collapse of glymphatic transport–which collectively accelerate amyloid pathology and cognitive decline in an age- and sex-dependent manner. By revealing the direct physical interaction between GPER and Aβ and the GPER-mediated metabolic-proteostatic axis in pericytes, this work highlights GPER as a promising therapeutic target. These results suggest that GPER-based interventions could rejuvenate the aging NVU and provide a non-hormonal strategy to combat neurodegeneration in Alzheimer’s disease.

## Supplementary Material

This is a list of supplementary files associated with this preprint. Click to download.

- manuscriptstable.docx
- Text.docx
- manuscriptsupfigure.pdf
- RNASeqmoduleinformation..xlsx
- AdditionalFile3.UncroppedBlots.pptx

## Data Availability

The data that support the findings of this study are available from the corresponding authors upon reasonable request.

## Abbreviations

Aβ: Amyloid-beta
AD: Alzheimer’s disease
GPER: G protein-coupled estrogen receptor
APP/PS1: Amyloid precursor protein/presenilin 1
AQP4: Aquaporin-4
BBB: Blood-brain barrier
BM: Basement membrane
CBF: Cerebral blood flow
Co-IP: Co-immunoprecipitation
DEG: Differentially expressed gene
E2: 17β-estradiol
GPER-KO: GPER-knockout
IP-MS: Immunoprecipitation-mass spectrometry
LRP1: Low-density lipoprotein receptor-related protein 1
LSCI: Laser Speckle Contrast Imaging
NVC: Neurovascular coupling
NVU: Neurovascular unit
PDGFRβ: Platelet-derived growth factor receptor beta
PFA: Paraformaldehyde
RBC: Red blood cell
ROI: Region of interest
S1: Primary somatosensory cortex
SREBP: Sterol regulatory element-binding protein
TEM: Transmission electron microscopy
TPLSM: Two-photon laser scanning microscopy
